# Exposure to traumatic events, PTSD, and alcohol use: A comparative study in Japan by type of traumatic event and gender

**DOI:** 10.1002/pcn5.70163

**Published:** 2025-07-28

**Authors:** Yuriko Takagishi, Masaya Ito, Hironori Kuga, Masaru Horikoshi

**Affiliations:** ^1^ National Center for Cognitive Behavior Therapy and Research National Center of Neurology and Psychiatry Kodaira Japan; ^2^ Institute of Human Sciences University of Tsukuba Tsukuba Japan; ^3^ Faculty of Human Sciences Musashino University Koto‐ku Japan

**Keywords:** trauma exposure, traumatic event, posttraumatic stress disorder, alcohol use disorder, gender differences

## Abstract

**Aim:**

The association between alcohol use disorder (AUD) and posttraumatic stress disorder (PTSD) is well established. However, many studies in Japan have focused on trauma related to natural disasters, which differ from international studies that include several trauma types. We examined whether trauma exposure and PTSD are associated with AUD risk across diverse trauma types. Additionally, because gender differences in drinking patterns and AUD may vary by country, we explored whether findings from international research hold in a Japanese context.

**Methods:**

An online survey of 6180 Japanese adults included 25 items from the World Health Organization's traumatic event list, the PTSD Checklist‐5, and the CAGE Questionnaire. Of these, 5150 reported exposure to traumatic events, and 1030 reported no exposure.

**Results:**

Participants' mean age was 43.79 years, and 52.8% were women. The AUD group comprised more men (59.1%) than the non‐AUD group (44.1%). Logistic regression analyses showed that trauma exposure was associated with higher odds ratios (ORs) of AUD among individuals without PTSD (OR = 1.55, 95% confidence interval [CI]: 1.24–1.94) and among those with PTSD (OR = 2.02, 95% CI: 1.62–2.52) compared to those with no trauma exposure. Among women, several non‐natural‐disaster events showed a higher risk of AUD than that posed by natural disasters. In men, physical violence by a partner was associated with a higher risk.

**Conclusion:**

These findings highlight the pertinence of assessing trauma characteristics and gender when addressing PTSD and AUD. They may inform gender‐sensitive interventions and culturally relevant policies targeting individuals at risk for comorbid PTSD and AUD.

## INTRODUCTION

Posttraumatic stress disorder (PTSD) is a severe mental disorder that develops in response to stressful or life‐threatening events (i.e., traumatic events). PTSD frequently co‐occurs with other psychiatric conditions, with alcohol use disorder (AUD) being one of the most common comorbidities. For example, Debell et al. reported that the rate of alcohol misuse in patients with PTSD ranged from 9.8% to 61.3% and that PTSD co‐occurred in 2.0% to 63.0% of individuals with AUD.^1^ The co‐occurrence of PTSD and AUD is associated with worse health outcomes^2^, ^3^ and fewer treatment improvements.^4^

Previous studies have suggested the possibility of a bidirectional causal relationship between AUD and PTSD. The self‐medication model proposes the hypothesis that individuals with PTSD use substances such as alcohol to alleviate symptoms, which in turn leads to the onset of AUD.^5^ Conversely, some models assume that alcohol use increases the risk of exposure to traumatic events (high‐risk model) and increases susceptibility to PTSD (sensitivity model).^6^, ^7^ While several other models exist to explain the association between AUD and PTSD, evidence suggests that multiple pathways lead to comorbidity and that these pathways may differ depending on gender.^8^

Studies on the relationship between trauma exposure (i.e., exposure to potentially traumatic events) and alcohol use in Japan have primarily focused on large‐scale natural disasters. These studies have shown that the prevalence of alcohol‐related problems after natural disasters tends to be lower than the national average.^9^ Additionally, reports indicate that alcohol consumption decreased after a catastrophe.^10^ These findings differ from previous studies in other countries that suggest an association between trauma exposure and alcohol use.^11^, ^12^

The diversity in results regarding the relationship between trauma exposure and alcohol use may be explained by differences in the type of traumatic events or cultural backgrounds. For example, individuals who have experienced interpersonal trauma are more likely to develop AUD than those who have not.^11^, ^13^ McFarlane noted that not all types of events are associated with at‐risk drinking, with involvement in life‐threatening accidents, witnessing severe injury, rape, and being a victim of serious physical assault and other types of traumatic events associated with at‐risk drinking.^14^ In contrast, combat and natural disasters were not associated with at‐risk drinking. Considering the cultural background of Japan, in addition to the nature of traumatic events, there are social norms that implicitly expect self‐restraint,^15^ which may have led to spontaneous inhibition of alcohol consumption during disasters. In this study, we examined various types of traumatic events in addition to natural disasters to verify whether the relationship between trauma exposure and alcohol use differs depending on the type of traumatic event. This will clarify whether the results of previous studies in Japan are due to cultural differences or the types of traumatic events focused on. Our hypotheses were as follows: (1) trauma exposure is associated with AUD, and (2) the association with AUD varies depending on the type of traumatic event and is more strongly associated with interpersonal trauma than with natural disasters.

Furthermore, gender differences have been reported for both PTSD and AUD. Following trauma exposure, women are more likely than men to meet the diagnostic criteria for PTSD (odds ratio [OR] = 1.72, 95% confidence interval [CI] = 1.27–2.34).^16^ In contrast, AUD is more common in men than women.^17^ In Japan as well, the prevalence of AUD was higher in men (1.9%, 95% CI: 1.4%–2.3%) than in women (0.2%, 95% CI: 0.0%–0.6%).^18^ However, in studies examining the association between trauma exposure and PTSD in young adults, while men showed significantly higher alcohol consumption than women, avoidant coping was found to have a significant influence on alcohol consumption beyond gender effects.^19^ Hence, we hypothesized that while AUD is more common in men and PTSD is more common in women, an association between trauma exposure and AUD is found in both men and women.

## METHODS

### Participants and procedure

The data for this study were derived from the National Survey for Stress and Health, an online survey conducted using the panelist pool of Macromill Inc., Japan's most extensive survey company, utilizing a panel of 1,182,255 respondents considered to be representative of the Japanese population. All procedures were carried out in accordance with relevant laws and institutional guidelines and have been approved by the institutional review board of the National Center of Neurology and Psychiatry (Approval Number: A2015‐086). The survey targeted clinical, subclinical, and non‐clinical populations and collected anonymous responses. For the clinical and subclinical population, to ensure an adequate sample size for PTSD, “disease panelists” who claimed to be currently undergoing treatment or to have undergone treatment in the past for specific conditions were selected. In this study, in addition to the PTSD panel, disease panelists with depressive disorders, anxiety disorders, or neurotic disorders that have a high comorbidity rate with PTSD were extracted. No stratified sampling based on demographics such as age, gender, or residence was conducted.

**Figure 1:**
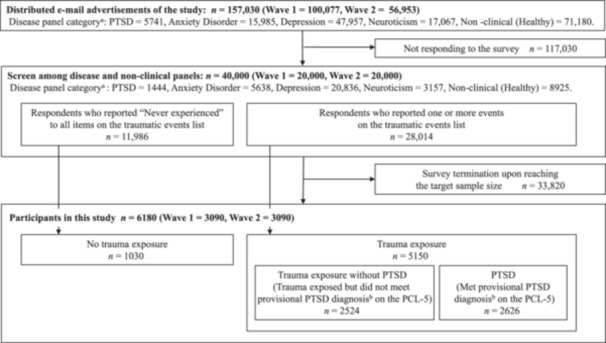
Participant recruitment flow. The flowchart illustrates participants’ recruitment and selection process in the National Survey for Stress and Health, including distributing advertisement emails, panel composition, trauma exposure screening, and final sample categorization based on trauma exposure and provisional posttraumatic stress disorder (PTSD) diagnosis using DSM‐5 criteria. *Note*: PCL‐5, Posttraumatic Stress Disorder Checklist for DSM‐5. ^a^Disease panel is defined by a positive answer for current or historical medical treatment status of each problem in annual surveys by the research company. ^b^Provisional DSM‐5 PTSD diagnoses were made using the DSM‐5 criteria, which require at least one item each from the B and C criteria and two items each from the D and E criteria.^23^, ^24^.

The flowchart for participant recruitment in this study is shown in Figure 1. The screening strategy was outlined in Ito et al.^20^ We surveyed two waves to control for seasonal effects. The Wave 1 survey was conducted in November 2016, and Wave 2 in March 2017. Advertisement emails were sent to 100,077 panelists in Wave 1 and 56,953 in Wave 2. Of these, 20,000 participated in the screening in Wave 1 and 20,000 in Wave 2. The screening was terminated when the target sample size was reached. The final sample included 5150 individuals who had been exposed to potentially traumatic events and 1030 individuals with no exposure to traumatic events. All participants read a full explanation of the study and provided informed consent by clicking the “agree” button before answering the questionnaire. The survey system automatically excluded participants with extremely short response times to improve data quality. The survey was designed so that participants could not proceed if they had not answered previous questions, ensuring that no data other than income was missing.

### Measures

#### Traumatic events

We used the traumatic event list from the PTSD module of the World Health Organization Composite International Diagnostic Interview, Version 3.0 (WHO‐CIDI).^21^ The WHO‐CIDI PTSD module includes 27 traumatic events. However, we excluded those items related to respondents' antisocial behavior per the survey company's guidelines: “Purposely injured, tortured, or killed someone,” “Accidentally caused serious injury or death,” and “Witnessed a physical fight at home.” We then added an “Other” category to the remaining 24 items, yielding 25 items. Lifetime experience was rated on a four‐point scale: 0 (*never experienced*), 1 (*experienced within the last month*), 2 (*experienced once more than 1 month ago*), and 3 (*experienced at least twice more than 1 month ago*).

#### PTSD caseness

We used the Japanese version of the Posttraumatic Stress Disorder Checklist for DSM‐5 (PCL‐5), a 20‐item assessment of PTSD symptoms.^22^ The reliability, precision, and validity of the Japanese version of this checklist were confirmed by Ito et al.^20^ Symptoms were rated on a 5‐point Likert scale (0 = *Not at all*, 4 = *Extremely*). Provisional PTSD cases were determined using the DSM‐5 criteria, requiring at least one symptom from clusters B and C and two from clusters D and E.^22^, ^23^, ^24^ We regarded a symptom as present if the participant rated it as two or higher. We divided individuals exposed to traumatic events into two groups: those meeting the criteria for PTSD using the PCL‐5 were classified as “PTSD,” and those not meeting the criteria were classified as “Trauma exposure without PTSD.”

#### Alcohol use disorder

We used the CAGE Questionnaire (CAGE), a 4‐item assessment of alcoholism.^25^ Compared to other screening tests, such as the Alcohol Use Disorders Identification Test (AUDIT), CAGE has fewer items, can be answered with less burden, and can be evaluated with the same cutoff point across countries and genders. Dhalla and Kopec^26^ discussed its reliability and validity. Kawakami et al.^27^ confirmed the reliability and validity of the Japanese version. The items included questions regarding the desire to reduce drinking, annoyance at being criticized for drinking, feeling guilty about drinking, and drinking in the morning to wake up. The respondents provided “yes/no” answers to these items. A total score of 2 or higher indicated AUD.

### Statistical analyses

First, we compared the participants' demographic characteristics using chi‐squared tests, depending on whether they had AUD. Next, we calculated the number and proportion of individuals with AUD by gender in the following groups: “No trauma exposure,” “Trauma exposure without PTSD,” and “PTSD.” Additionally, we calculated the number and proportion of individuals with AUD by gender for each traumatic event exposed. We subsequently conducted a series of logistic regression analyses to examine the following: (1) the OR of AUD in the presence or absence of trauma exposure and PTSD, and (2) the OR of AUD between types of traumatic events. Separate analyses were conducted for gender, and all models were adjusted for demographic factors. Specifically, adjustments were made for age, region of residence, employment, household income, marital status, smoking, and mental illness. These variables were selected based on prior research^28^ identifying them as predictors of AUD and on variables that showed differences between those with and without AUD in the present study, as described below. In the analysis by type of traumatic event, we followed Peduzzi et al.,^29^ who excluded variables with several events per variable of less than 10. For comparison with previous Japanese studies, natural disasters were set as the reference traumatic event. Statistical analyses were performed using the IBM SPSS Statistics Version 29.0.

## RESULTS

### Participants' characteristics

Table 1 summarizes the participants' demographic characteristics. The sample consisted of individuals from all 47 prefectures in Japan with a mean age of 43.79 years (standard deviation [SD] = 11.03). Of the participants, 52.8% (*n* = 3,260) were women. When comparing the AUD and non‐AUD groups, the AUD group had a higher proportion of men (AUD group = 59.1%, non‐AUD group = 44.1%). Differences between the two groups were also observed in all other demographic variables: The AUD group had a higher average age, fewer participants from the Chubu region and more from the Kyushu region, more employed individuals and fewer homemakers, higher income levels, higher marriage rates, and higher smoking rates. Additionally, the AUD group had a higher prevalence of all mental disorders examined compared to the non‐AUD group.

**Table 1:** Participants’ characteristics.

Characteristic	Non‐AUD (*n* = 4883)			AUD (*n* = 1297)			*p* values
	Mean (*n*)	SD (%)		Mean (*n*)	SD (%)		
Gender (male)	2154	44.1	%	766	59.1	%	<0.001
Age (years)	43.5	11.2		44.8	10.5		<0.001
Region							0.018
Hokkaido	298	6.1	%	65	5.0	%	
Tohoku	314	6.4	%	103	7.9	%	
Kanto	1631	33.4	%	446	34.4	%	
Chubu	904	18.5	%	206	15.9	%	
Kinki	936	19.2	%	240	18.5	%	
Chugoku	231	4.7	%	54	4.2	%	
Shikoku	109	2.2	%	29	2.2	%	
Kyushu	460	9.4	%	154	11.9	%	
Employment							<0.001
Employed	2,941	60.2	%	853	65.8	%	
Homemaker	840	17.2	%	167	12.9	%	
Other	372	7.6	%	92	7.1	%	
Unemployed	730	14.9	%	185	14.3	%	
Household income							<0.001
≤3.9 million yen	2007	41.1	%	506	39.0	%	
4–7.9 million yen	1504	30.8	%	413	31.8	%	
≥8 million yen	580	11.9	%	213	16.4	%	
Don't know	425	8.7	%	97	7.5	%	
No data	367	7.5	%	68	5.2	%	
Marital status (married)	2316	47.4	%	680	52.4	%	0.001
Habitual use of alcohol (+)	1381	28.3	%	1026	79.1	%	<0.001
Habitual use of tobacco (+)	1301	26.6	%	622	48.0	%	<0.001
Mental disorder							
Major depressive disorder	2296	47.0	%	664	51.2	%	0.007
Obsessive–compulsive disorder	544	11.1	%	287	22.1	%	<0.001
Social anxiety disorder	634	13.0	%	293	22.6	%	<0.001
Panic disorder	837	17.1	%	324	25.0	%	<0.001
Psychotic disorder	381	7.8	%	238	18.4	%	<0.001
Bipolar disorder	373	7.6	%	237	18.3	%	<0.001
Eating disorder	320	6.6	%	217	16.7	%	<0.001
Generalized anxiety disorder	607	12.4	%	291	22.4	%	<0.001
Dysthymia	363	7.4	%	250	19.3	%	<0.001
Current other mental disorder	1107	22.7	%	424	32.7	%	<0.001

*Note*: *p* values were calculated based on Pearson's χ^2^ tests. This table summarizes the participants’ background characteristics, including age, gender, region of residence, employment status, household income, marital status, smoking status, and prevalence of comorbid mental disorders, compared between individuals with and without AUD.

Abbreviations: AUD, alcohol use disorder; SD, standard deviation.

Table 2 shows the number and proportion of individuals with AUD by trauma exposure and PTSD status and by gender for each type of traumatic event. For several events, including war and sexual violence against men, the number of AUD cases was less than 10. Among men, the highest AUD prevalence rates were in those with experience of “rape” (58.3%), “being beaten by a spouse or romantic partner”(54.5%), “combat experience” (50.0%), and “working as a relief worker in a war zone” (50.0%). Among women, the highest AUD prevalence rates were for those who had been a “relief worker in a war zone” (100.0%), a “civilian in region of terror” (66.7%), and those “beaten up by spouse or romantic partner” (27.3%).

**Table 2:** Prevalence of AUD for trauma exposure, PTSD, and traumatic events.

	All				Men				Women			
	Total (*n* = 6180)	AUD (*n* = 1297)	Prevalence of AUD		Total (*n* = 2920)	AUD (*n* = 766)	Prevalence of AUD		Total (*n* = 3260)	AUD (*n* = 531)	Prevalence of AUD	
Trauma exposure and PTSD caseness												
No trauma exposure	1030	139	13.5	%	503	90	17.9	%	527	49	9.3	%
Trauma exposure without PTSD	2524	475	18.8	%	1233	283	23.0	%	1291	192	14.9	%
PTSD	2626	683	26.0	%	1184	393	33.2	%	1442	290	20.1	%
Type of traumatic event												
Accident												
Natural disaster	364	73	20.1	%	196	57	29.1	%	168	16	9.5	%
Toxic chemical exposure	8	2	25.0	%	5	2	40.0	%	3	0	0.0	%
Automobile accident	697	161	23.1	%	480	133	27.7	%	217	28	12.9	%
Other life‐threatening accident	62	20	32.3	%	41	17	41.5	%	21	3	14.3	%
Man‐made disaster	84	23	27.4	%	62	18	29.0	%	22	5	22.7	%
Life‐threatening illness	370	79	21.4	%	235	62	26.4	%	135	17	12.6	%
War events												
Combat experience	6	3	50.0	%	6	3	50.0	%	0	0	—	
Relief worker in war zone	4	3	75.0	%	2	1	50.0	%	2	2	100.0	%
Civilian in war zone	4	1	25.0	%	3	1	33.3	%	1	0	0.0	%
Civilian in region of terror	6	3	50.0	%	3	1	33.3	%	3	2	66.7	%
Refugee	2	0	0.0	%	1	0	0.0	%	1	0	0.0	%
Saw atrocities	23	5	21.7	%	12	4	33.3	%	11	1	9.1	%
Physical violence												
Kidnapped	11	2	18.2	%	3	0	0.0	%	8	2	25.0	%
Beaten up by caregiver	657	127	19.3	%	215	58	27.0	%	442	69	15.6	%
Beaten up by spouse or romantic partner	370	113	30.5	%	44	24	54.5	%	326	89	27.3	%
Beaten up by someone else	349	86	24.6	%	243	71	29.2	%	106	15	14.2	%
Mugged or threatened with a weapon	69	18	26.1	%	41	10	24.4	%	28	8	28.6	%
Sexual violence												
Raped	84	21	25.0	%	12	7	58.3	%	72	14	19.4	%
Sexually assaulted	179	39	21.8	%	21	8	38.1	%	158	31	19.6	%
Stalked	160	35	21.9	%	28	9	32.1	%	132	26	19.7	%
Death												
Unexpected death of loved one	636	150	23.6	%	295	80	27.1	%	341	70	20.5	%
Network events												
Child with serious illness	118	22	18.6	%	53	13	24.5	%	65	9	13.8	%
Traumatic event to loved one	429	86	20.0	%	189	49	25.9	%	240	37	15.4	%
Witnessed death/dead body or serious injury	243	56	23.0	%	138	36	26.1	%	105	20	19.0	%
Other	215	30	14.0	%	89	12	13.5	%	126	18	14.3	%

*Note*: This table shows the number and percentage of individuals with AUD, stratified by trauma exposure, PTSD diagnosis, and gender, for each category of traumatic events. Due to small sample sizes, caution is advised when interpreting cells with fewer than 10 cases.

Abbreviations: AUD, alcohol use disorder; PTSD, posttraumatic stress disorder.

### Association between trauma exposure, PTSD, and AUD

Table 3 presents the associations between trauma exposure, PTSD, and AUD. The reference group included those with no trauma exposure. We compared this group with those with trauma exposure without PTSD and those with PTSD. AUD was significantly more common among those who had been exposed to trauma (18.8% without PTSD and 26.0% with PTSD) than among those who had not (13.5%). These findings were consistent across the genders.

**Table 3:** Odds of AUD for trauma exposure and PTSD.

Trauma exposure and PTSD caseness	All^a^			Men^b^			Women^b^		
	OR	(95% CI)	*p* value	OR	(95% CI)	*p* value	OR	(95% CI)	*p* value
No trauma exposure	1	(Reference)		1	(Reference)		1	(Reference)	
Trauma exposure without PTSD	1.55	(1.24–1.94)	<0.001	1.48	(1.11–1.98)	0.007	1.69	(1.18–2.44)	0.005
PTSD	2.02	(1.62–2.52)	<0.001	2.04	(1.54–2.71)	<0.001	2.09	(1.47‐2.99)	<0.001

*Note*: This table presents the prevalence of AUD in groups with no trauma exposure, trauma exposure without PTSD, and trauma exposure with PTSD. Results are shown for the overall sample and separately by gender.

Abbreviations: AUD, alcohol use disorder; CI, confidence interval; OR, odds ratio; PTSD, posttraumatic stress disorder.

^a^ Logistic regression, adjusted for age, gender, region, employment, household income, marital status, smoking, and mental disorders.

^b^ Logistic regression, adjusted for age, region, employment, household income, marital status, smoking, and mental disorders.

### Association between the type of traumatic events and AUD

In Table 4, we report the ORs for AUD among individuals exposed to different types of traumatic events. The reference category was set as natural disasters.

**Table 4:** Odds of AUD for type of traumatic event exposed.

Type of traumatic event	All^a^, ^b^			Men^c^, ^d^			Women^c^, ^e^		
	OR	(95% CI)	*p* value	OR	(95% CI)	*p* value	OR	(95% CI)	*p* value
Accident									
Natural disaster	1	(Reference)		1	(Reference)		1	(Reference)	
Automobile accident	1.15	(0.81–1.61)	0.434	0.97	(0.65–1.45)	.882	1.50	(0.75–3.01)	0.252
Other life‐threatening accident	1.68	(0.87–3.24)	0.122	1.62	(0.76–3.48)	.213	—	—	—
Man‐made disaster	1.45	(0.81–2.59)	0.212	1.12	(0.57–2.20)	.743	—	—	—
Life‐threatening illness	1.15	(0.78–1.70)	0.475	0.93	(0.59–1.49)	.773	1.78	(0.83–3.81)	0.139
Physical violence									
Beaten up by caregiver	1.17	(0.82–1.66)	0.387	0.98	(0.61–1.56)	0.924	1.82	(0.98–3.37)	0.057
Beaten up by spouse or romantic partner	2.47	(1.68–3.62)	<0.001	3.21	(1.53–6.74)	0.002	3.50	(1.89–6.45)	<0.001
Beaten up by someone else	1.25	(0.85–1.85)	0.262	1.12	(0.71–1.77)	0.625	1.53	(0.67–3.51)	0.314
Mugged or threatened with a weapon	1.38	(0.72–2.63)	0.335	0.83	(0.36–1.92)	0.665	—	—	—
Sexual violence									
Raped	1.60	(0.85–3.00)	0.148	—	—	—	2.21	(0.95–5.17)	0.067
Sexually assaulted	1.71	(1.05–2.76)	0.029	—	—	—	2.56	(1.28–5.11)	0.008
Stalked	1.62	(0.99–2.65)	0.056	—	—	—	2.47	(1.21–5.03)	0.013
Death									
Unexpected death of loved one	1.36	(0.97–1.93)	0.076	1.00	(0.65–1.55)	0.983	2.54	(1.37–4.72)	0.003
Network events									
Child with serious illness	1.11	(0.63–1.96)	0.715	0.80	(0.38–1.68)	0.551	—	—	—
Traumatic event to loved one	1.25	(0.85–1.83)	0.251	1.03	(0.64–1.67)	0.901	1.92	(0.99–3.75)	0.055
Witnessed death/dead body or serious injury	1.25	(0.81–1.91)	0.310	0.87	(0.51–1.49)	0.616	2.54	(1.19–5.40)	0.016

*Note*: This table displays the results of logistic regression analyses examining the association between exposure to specific types of traumatic events and the likelihood of AUD. Natural disasters were used as the reference category. Analyses were conducted for the overall sample and separately by gender.

Abbreviations: AUD, alcohol use disorder; CI, confidence interval; OR, odds ratio.

^a^ Logistic regression, adjusted for age, gender, region, employment, household income, marital status, smoking, and mental disorders.

^b^ Exclude toxic chemical exposure, kidnapped, war events, and other from analysis.

^c^ Logistic regression, adjusted for age, region, employment, household income, marital status, smoking, and mental disorders.

^d^ Exclude toxic chemical exposure, kidnapped, war events, sexual violence and other from analysis.

^e^ Exclude toxic chemical exposure, other life‐threatening accident, man‐made disaster, war events, kidnapped, mugged or threatened with a weapon, child with serious illness, and other from analysis.

The results of the logistic regression analysis showed that the OR for AUD was higher in those “beaten up by spouse or romantic partner” and those “sexually assaulted” among all respondents (OR = 2.47, 95% CI: 1.68–3.62; OR = 1.71, 95% CI: 1.05–2.76, respectively). Among women, in addition to the above, the OR in the death category was higher in those who had been “stalked,” and those who had experienced “unexpected death of loved one” or “witnessed death/dead body or serious injury” (OR = 2.47, 95% CI: 1.21–5.03; OR = 2.54, 95% CI: 1.37–4.72; OR = 2.54, 95% CI: 1.19–5.40, respectively). Among men, only those who had been “beaten up by spouse or romantic partner” had a significantly higher OR for AUD than those who had experienced natural disasters (OR = 3.21, 95% CI: 1.53–6.74).

## DISCUSSION

This study examined the relationship between PTSD and AUD in Japan, focusing on trauma type and gender differences. The findings partially support our hypotheses: Both trauma exposure and PTSD are associated with the risk of AUD in men and women. Moreover, AUD risk varied by trauma type, with gender‐difference‐specific patterns: Among men, being beaten up by a spouse or romantic partner carried a higher risk than that carried by natural disasters; among women, several non‐natural‐disaster events showed a higher AUD risk.

A notable feature of our sample was a higher AUD prevalence than that in previous Japanese studies of natural‐disaster‐affected regions^9^, ^30^ or national surveys.^20^ To recruit sufficient participants with PTSD, we used a disease panel that included PTSD, anxiety disorders, depression, and neurotic disorders. Because AUD commonly co‐occurs with various mental disorders, this sampling strategy may have inflated AUD rates. Therefore, caution is needed when generalizing these results to the broad Japanese population. Despite these limitations, the purpose of this study was to investigate the association between trauma exposure, PTSD, and AUD across various traumatic events, to identify high‐risk groups, and inform prevention and early intervention strategies.

Logistic regression analysis revealed that trauma‐exposed individuals had higher odds of AUD than those unexposed individuals (without PTSD: OR = 1.55, 95% CI: 1.24–1.94; with PTSD: OR = 2.02, 95% CI: 1.62–2.52). This Japanese survey also confirmed the PTSD–AUD association observed in prior research. These findings differ from previous Japanese studies,^9^, ^10^ likely because we examined a broader range of traumatic events. We found that being beaten by a spouse or romantic partner and sexually assaulted were more strongly associated with AUD risk than natural disasters, consistent with international data.^11^ First, alcohol access is uniquely restricted during disasters: supply disruptions, reduced income, and reconstruction priorities often lower consumption temporarily. Such constraints may explain the temporary decrease in alcohol consumption after a disaster. Second, Japanese social norms implicitly expect self‐restraint in collective settings (e.g., evacuation centers), where excessive drinking may be considered deviant behavior. Conversely, traumas such as sexual assault or partner violence are private experiences, less visible to the community. Coping styles may differ: sexual assault often induces shame, isolation, and helplessness, prompting some individuals to use alcohol to manage these emotions.^31^ Unlike shared disaster experiences, personal traumas may engender distinct drinking patterns.

This study extends prior Japanese research on AUD in disaster contexts by examining diverse traumatic events and comparing their associations with AUD, demonstrating that the strength of the PTSD–AUD relationship varies by trauma type.

For both men and women, being “beaten up by spouse or romantic partner” had a higher AUD risk than that carried by natural disasters. However, even within the physical violence category, risk varied: Events such as “beaten by a caregiver,” “beaten up by someone else,” and “threatened or attacked with a weapon” did not show uniformly high AUD risk. Therefore, when examining trauma–AUD associations, it is pertinent to consider not only the form of harm (e.g., physical violence) but also the qualitative context of each event.

The association between specific traumatic events and AUD risk also differed by gender. In women, direct perpetrator harm (being beaten up by a spouse or romantic partner, sexual assault, and stalking), sudden death of a loved one, and witnessing death or serious injury were all linked to higher AUD risk. These findings align with prior studies^32^ highlighting gender differences in trauma responses and underscore the need for detailed, gender‐sensitive analyses.

In men, only being beaten by a spouse or partner showed a significantly higher AUD risk compared to natural disasters. This suggests that victimization continuously experienced during a period of adulthood is particularly relevant to alcohol use in men. Although not formally analyzed because of limited sample size, some war‐related events and sexual violence events also exhibited high AUD proportions. Even low‐prevalence traumas in Japan may carry substantial AUD risk and warrant attention regardless of frequency.

This study has some limitations. First, its cross‐sectional data design precludes causal inference: Although longitudinal studies (e.g., Livingston et al.^33^) suggest that PTSD can lead to AUD, others report the reverse relationship,^34^ so AUD may have exacerbated participants' PTSD. Second, some trauma categories had insufficient sample sizes for robust subgroup analyses, and certain CIDI event items were omitted due to survey constraints. Third, participants were limited to Internet users registered with the survey company, which may restrict generalizability. Fourth, we used the CAGE Questionnaire to screen for AUD; while it is a valuable screening tool, its sensitivity has been reported to be low in women.^26^ In a national survey in Japan,^18^ the prevalence of AUD using the CAGE Questionnaire was nearly twice that of the AUDIT among women, while similar regardless of the screening tool used among men. The high prevalence of AUD in this study may have been influenced by the measurement tools. Future studies should employ longitudinal designs, recruit broad populations, and incorporate detailed and accurate measurements to address these limitations.

## CONCLUSION

Exposure to traumatic events and PTSD are associated with a higher risk of AUD regardless of gender. This association varies depending on the type of traumatic event, and the results of this study highlight the importance of considering both the type and quality of the experience when developing prevention and intervention strategies. Even events with low occurrence rates showed a high proportion of AUD patients, indicating that focusing solely on high‐frequency events is not appropriate when considering prevention and intervention for AUD.

## AUTHOR CONTRIBUTIONS

**Yuriko Takagishi**: Conceptualization; formal analysis; funding acquisition; methodology; writing—original draft; writing—review and editing. **Masaya Ito**: Conceptualization; data curation; methodology; project administration; writing—review and editing. **Hironori Kuga**: Writing—review and editing. **Masaru Horikoshi**: Funding acquisition; writing—review and editing.

## CONFLICT OF INTEREST STATEMENT

All procedures were carried out in accordance with relevant laws and institutional guidelines and have been approved by the institutional review board of the National Center of Neurology and Psychiatry.

## ETHICS APPROVAL STATEMENT

All procedures were carried out in accordance with relevant laws and institutional guidelines and have been approved by the institutional review board of the National Center of Neurology and Psychiatry (Approval Number: A2015‐086).

## PATIENT CONSENT STATEMENT

All participants read a full explanation of the study and provided informed consent.

## CLINICAL TRIAL REGISTRATION

N/A.

## Data Availability

The data that support the findings of this study are available from the corresponding author, Masaya Ito, upon reasonable request.
